# Investigation of Urinary Sestrin2 in Patients with Obstructive Sleep Apnea

**DOI:** 10.1007/s00408-019-00205-8

**Published:** 2019-02-15

**Authors:** Lu Bai, Chunying Sun, Huifen Zhai, Chen Chen, Xiaotian Hu, Xiulin Ye, Min Li, Yan Fang, Weimin Yang, Haoyan Wang, Shibo Sun

**Affiliations:** 1 Department of Respiratory and Critical Care Medicine, First Affiliated Hospital, Kunming Medical University, Kunming, China; 2 2015 Innovation Class, Kunming Medical University, Kunming, China; 3 Obstetrical Department, Zhengzhou Maternity and Child Care Center, Zhengzhou, China; 4 Department of Respiratory Medicine, Beijing Friendship Hospital, Capital Medical University, Beijing, China; 5 School of Pharmaceutical Science & Yunnan Key Laboratory of Pharmacology for Natural Products, Kunming Medical University, Kunming, China

**Keywords:** Sestrin2, Obstructive sleep apnea, Apnea/hypopnea index, High-density lipoprotein

## Abstract

**Background:**

Obstructive sleep apnea (OSA) is a disease seriously threatening individual health, which results in serious complications such as hypertension and stroke. These complications are associated with oxidative stress triggered by intermittent hypoxia in OSA. Sestrin2 is a crucial factor involved in oxidative stress. The goal of this study was to investigate if a relationship exists between OSA and Sestrin2.

**Methods:**

We prospectively enrolled 71 subjects, and 16 patients of them with severe OSA completed 4 weeks of nasal continuous positive airway pressure (nCPAP) therapy. We measured and compared the concentration of Sestrin2 in the urine of all subjects, as well as the changes between before and after nCPAP treatment. Additionally, the correlation between Sestrin2 and sleep parameters was analyzed, and the multiple linear regression analysis with stepwise selection was performed to explore the relationship between Sestrin2 and various factors.

**Results:**

A total of 71 subjects were enrolled and divided into two groups: OSA group (*n* = 41), control group (*n* = 30). The level of urinary Sestrin2 in OSA patients was significantly higher than that of the control group, and increased with the severity of OSA, while it reduced after nCPAP treatment. Additionally, Sestrin2 was positively correlated with apnea/hypopnea index (AHI), oxygen desaturation index, oxygen saturation < 90% percentage of recording time spent (PRTS) and high-density lipoprotein (HDL), while negatively correlated with the lowest oxygen saturation. Importantly, Sestrin2 was independently associated with AHI, oxygen saturation < 90% PRTS and HDL.

**Conclusions:**

Urinary Sestrin2 is involved in OSA, and is a paramount marker of OSA severity.

## Introduction

Obstructive sleep apnea (OSA) is a shared sleep-related disorder. According to the survey, the estimated prevalence of OSA is 0.7–3.3% [1–3]. It is characterized by periodic recurrent episodes of upper airway obstruction during sleep, resulting in apnea [4], and the occurrence of OSA is capable of disrupting sleep integrity, impairing ventilation, and resulting in intermittent hypoxia [5], which seriously lead to coronary artery disease, high blood pressure, cerebrovascular accident, stroke, etc. [6]. There is increasing evidence that these complications are closely related to oxidative stress triggered by intermittent hypoxia [6–9].

Sestrin, originally discovered as p53-inducible proteins, is a highly conserved family of proteins that are thought to protect cells from various damages [10]. The Sestrin family has three distinct members in mammals, which include Sestrin1, Sestrin2, and Sestrin3 [11]. Sestrin2, among the three members, also known as hypoxia-inducible gene (Hi95), is an acute response protein that plays a paramount role in various conditions such as excessive oxidative stress, hypoxia, DNA damage, etc. [12]. Moreover, Sestrin2 expression is obviously up-regulated in oxidative stress-related diseases, exerting anti-oxidation, preventing the production of reactive oxygen species (ROS), reducing the accumulation of ROS as an inducible factor of oxidation, and thus protecting cells from oxidative stress [13, 14]. Chronic intermittent hypoxia (CIH) occurs in patients with OSA during sleeping [15], leading to a strong oxidative stress response [16]. Accordingly, we speculated that Sestrin2 might be involved in the OSA. To study the relationship between Sestrin2 and OSA may be beneficial in discovering the reasons of oxidative stress-related complications in OSA patients. However, so far, we have not found any relevant reports. The goal of this study was to investigate the changes in urinary Sestrin2 concentration in OSA patients.

## Method

### Research Population

We prospectively enrolled 71 subjects who were entirely performed by polysomnography (PSG) tests because of snoring, including 41 newly diagnosed OSA patients and 30 healthy controls at the First Affiliated Hospital of Kunming Medical University, from June 2016 to June 2018. The inclusion criteria were patients with 18–65 years old, and the exclusion criteria were patients with heart failure, lung disease, cerebrovascular disease, kidney disease, metabolic diseases like diabetes, and the OSA patients who have been treated before this study.

All patients underwent a systematic physical examination prior to PSG testing to measure the height, weight, waist-to-hip ratio, and neck circumference (NC). Their body mass index (BMI) was calculated. The Epworth Sleepiness Score (ESS) was used to assess the degree of daytime sleepiness. This study was approved by the Ethics Committee of the First Affiliated Hospital of Kunming Medical University, and the patient’s informed consents were obtained.

### PSG

All subjects were examined by overnight PSG (Alice 5, American) and recorded parameters including EEG, ECG, oral and nasal airflow, oxygen saturation, and chest and abdomen movements. Apnea was defined as a respiratory amplitude reduction ≥ 90% for more than 10 s. Hypopnea was defined as a decrease in respiratory airflow amplitude reduction ≥ 30% with a decrease in oxygen saturation ≥ 3% or arousal [17]. All PSG records were analyzed by two experts to obtain apnea/hypopnea index (AHI). Normal subject was defined with AHI < 5, OSA was defined with AHI ≥ 5. The hypoxemia index was indicated by oxygen saturation < 90% percentage of recording time spent (PRTS).

### Pressure Titration Under PSG

An overnight pressure titration was performed under PSG testing using an auto-continuous positive airway pressure (Auto-CPAP) machine (Philips-Respironics REMstarAuto). The airflow signal was collected by the mask pressure signal. The humidifier was routinely used during titration. If the PSG result demonstrated AHI ≤ 5/h, 90th percentile recording pressure was defined as the titration pressure.

### nCPAP Treatment

Patients enrolled in the treatment response analysis were treated with a nasal CPAP (nCPAP) machine (Resmed, S9 Escape, Australia). There was a data card on each machine to record the application time. We read the data card and downloaded the data after 4 weeks of treatment. Patients who were treated for more than 4 h/day were included in the treatment analysis.

### Urine Test

5–7 mL urine specimens were collected from the patients on an empty stomach in the morning after the PSG test and 4 weeks nCPAP treatment, then centrifugated 20 min by 3000 rpm at 4 °C. Urine supernatant was extracted and stored in − 80 °C refrigerator for testing. The urinary Sestrin2 concentration was measured with an ELISA kit (Mlbio, Shanghai, China). Other tests including fasting blood glucose, cholesterol, low-density lipoprotein (LDL), high-density lipoprotein (HDL), and urine creatinine were tested by the First Affiliated Hospital of Kunming Medical University. Sestrin2 was adjusted with creatinine: adjust Sestrin2 = (Sestrin2/creatinine) × 10^4^.

### Statistical Analysis

Data were presented as mean ± standard derivation. In addition, the single-sample Kolmogorov–Smirnov method was used to detect whether the data were normally distributed. Normally distributed data were compared by an unpaired *t*-test, and the data between before and after treatment were compared with paired *t*-test. Rank sum test was used for non-normally distributed data. Pearson’s correlation analysis was applied for correlation analysis. Linear stepwise regression analysis was used to detect the relationship between Sestrin2 and other factors (age, gender, waist-to-hip, BMI, NC, AHI, mean oxygen saturation, lowest oxygen saturation, oxygen saturation < 90% PRTS, ESS, TG, TC, LDL, HDL, fasting blood glucose).

## Result

71 Subjects were prospectively enrolled in this study, and were divided into two groups: OSA group (39.29 ± 7.73 years old, *n* = 41) and control group (38.70 ± 8.86 years old, *n* = 30). All patient characteristics and the results of urinary Sestrin2 were listed in Table 1. OSA patients were divided into three subgroups according to AHI: mild group 5 ≤ AHI < 15, moderate group 15 ≤ AHI < 30, and severe group AHI ≥ 30. Sixteen patients with severe OSA completed 4 weeks nCPAP treatment, and the treatment time was greater than 4 h/day, and then the levels of urinary Sestrin2 after treatment were measured.

**Table 1:** Demographics, sleep profiles, blood and urinary measurements of the OSA and control groups

	Control group (*n* = 30)	OSA group (*n* = 41)	*P* values
Age (years)	38.70 ± 8.86	39.29 ± 7.73	0.765
Gender (male/female)^a^	20/10	32/9	0.416
BMI (kg/m^2^)	27.09 ± 4.22	28.55 ± 4.62	0.176
Neck circumference (cm)	36.06 ± 5.47	38.56 ± 6.90	0.093
Waist-to-hip ratio	0.88 ± 0.07	0.92 ± 0.08	0.022*
AHI (events/h)	2.32 ± 1.20	35.00 ± 20.86	< 0.001*
ODI (events/h)	1.59 ± 0.90	31.17 ± 19.31	< 0.001*
Lowest oxygen saturation (%)	90.48 ± 0.99	79.93 ± 8.28	< 0.001*
Mean oxygen saturation (%)	93.50 ± 0.94	90.40 ± 2.91	< 0.001*
Oxygen saturation < 90% PRTS (%)	0.46 ± 1.42	19.35 ± 8.92	< 0.001*
Total sleep time (min)	316.8 ± 38.71	329.83 ± 40.71	0.179
Arousal index (events/h)	13.17 ± 7.32	35.86 ± 20.63	< 0.001*
Stage of REM (%)	13.15 ± 3.79	11.36 ± 3.25	0.035*
Stage of NREM (%)	86.85 ± 3.79	88.64 ± 3.25	0.035*
Triglycerides (mmol/L)	1.51 ± 0.61	1.77 ± 1.12	0.224
Sestrin2 (ng/mL creatinine)	2.69 ± 1.11	3.39 ± 1.65	0.035*
Total cholesterol (mmol/L)	4.08 ± 0.80	4.31 ± 0.64	0.180
Low-density lipoprotein (mmol/L)	2.31 ± 0.63	2.51 ± 0.58	0.160
High-density lipoprotein (mmol/L)	1.03 ± 0.29	0.99 ± 0.24	0.532
Fasting blood glucose (mmol/L)	4.31 ± 0.53	4.56 ± 0.56	0.064
ESS (scores)	6.73 ± 2.46	11.92 ± 4.30	< 0.001*

Values are indicated as the mean ± standard deviation

*BMI* body mass index, *AHI* apnea/hypopnea index, *ODI* oxygen desaturation index, *PRTS* percentage of recording time spent, *REM* rapid eye movement, *NREM* non-rapid eye movement, *ESS* Epworth Sleepiness Score

*P < 0.05, the results are statistically significant

^a^Values are indicated as numbers

**Fig. 1:**
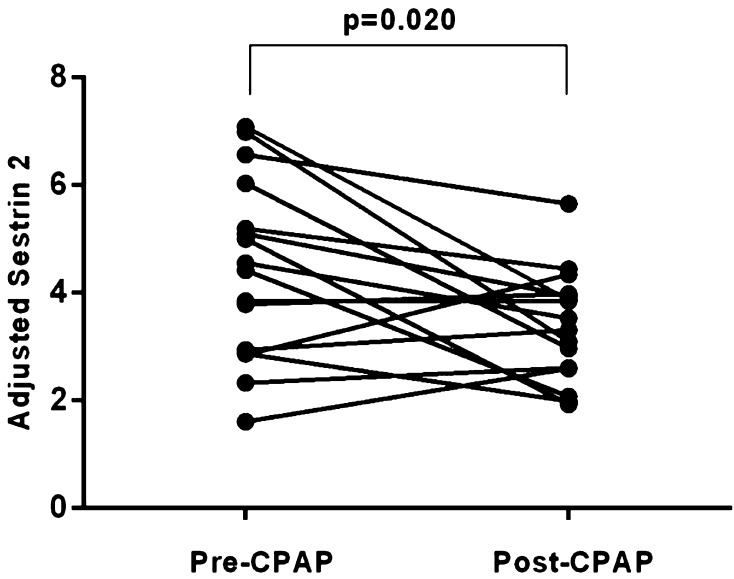
Results of adjusted Sestrin2 level of 16 severe OSA before and after CPAP treatment

**Fig. 2:**
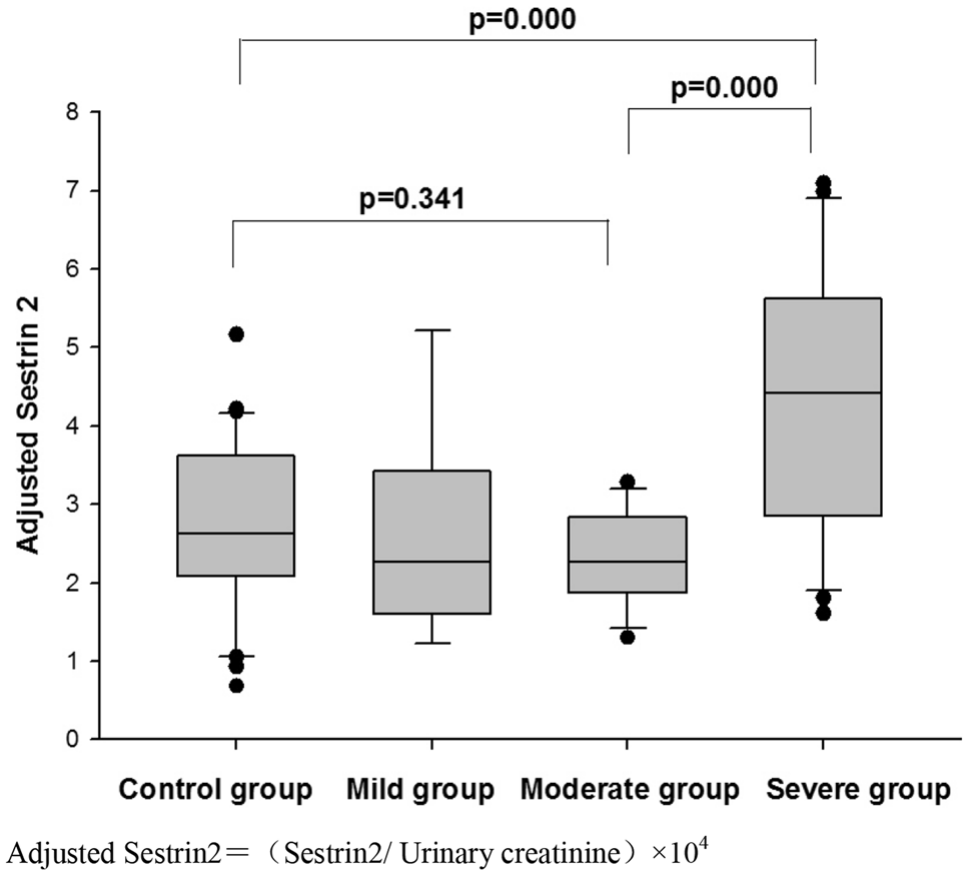
Adjusted Sestrin2 levels of control group and OSA subgroups. Adjusted Sestrin2 = (Sestrin2/urinary creatinine) × 10^4^

The level of urinary Sestrin2 in the OSA group was significantly higher than that of the control group (*P* = 0.035), and as the severity of OSA increased, Sestrin2 level also increased. In addition, Sestrin2 was significantly reduced after nCPAP treatment (Fig. 1). Subgroup analysis demonstrated that the concentration of Sestrin2 in the severe OSA group was significantly higher than that of the other subgroups and control group (Fig. 2).

Sestrin2 was positively correlated with AHI, oxygen desaturation index (ODI), oxygen saturation < 90% PRTS, HDL, fasting blood glucose, and OSA severity, but negatively correlated with lowest oxygen saturation. Importantly, these relationships still existed after adjusting for age, gender, waist-to-hip, NC, and BMI (Table 2).

**Table 2:** Spearman’s correlations between adjusted Sestrin2 and the other factors

	*r*	*P* values	*r* ^a^	*P* values
Age	0.170	0.156		
BMI	0.075	0.535		
Neck circumference	−0.043	0.725		
Waist-to-hip ratio	0.073	0.545		
AHI	0.376	0.001*	0.566	< 0.001*
ODI	0.314	0.008*	0.559	< 0.001*
Lowest oxygen saturation	−0.358	0.002*	−0.350	0.004*
Mean oxygen saturation	−0.160	0.183	−0.138	0.268
Oxygen saturation < 90% PRTS	0.378	0.001*	0.562	< 0.001*
Total sleep time	−0.047	0.698	0.044	0.726
Arousal index	−0.003	0.979	0.215	0.083
Stage of REM	0.070	0.564	−0.016	0.901
Stage of NREM	−0.070	0.564	0.016	0.901
Triglycerides	−0.054	0.654	−0.030	0.814
Total cholesterol	0.029	0.811	0.084	0.505
Low-density lipoprotein	0.001	0.996	−0.012	0.922
High-density lipoprotein	0.344	0.003*	0.325	0.008*
Fasting blood glucose	0.305	0.010*	0.274	0.026*
Epworth Sleepiness Score	0.298	0.011*	0.259	0.036*
Severity of OSA	0.405	0.000*	0.388	0.001*

*BMI* body mass index, *AHI* apnea/hypopnea index, *ODI* oxygen desaturation index, *PRTS* percentage of recording time spent, *REM* rapid eye movement, *NREM* non-rapid eye movement, *OSA* obstructive sleep apnea

*P < 0.05, the results are statistically significant

^a^Adjusting for age, BMI, gender, waist-to-hip ratio, neck circumference

The multiple linear regression analysis with stepwise selection showed that Sestrin2 was associated with AHI, oxygen saturation < 90% PRTS, and HDL (Table 3). However, there was no association between Sestrin2 and waist-to-hip ratio.

**Table 3:** Stepwise multiple regression model of adjusted urinary Sestrin2 levels in the OSA group (adjusted *R*^2^ = 0.745)

	*B* (SE)	*β*	*P* values
Constant	−1.078 (0.525)		0.047*
AHI	0.033 (0.007)	0.420	< 0.001*
Oxygen saturation < 90% PRTS	0.094 (0.016)	0.509	< 0.001*
HDL	1.435 (0.444)	0.262	0.003*

Independent variables considered: age, gender, BMI, waist-to-hip ratio, neck circumference, AHI, lowest oxygen saturation, oxygen saturation < 90% PRTS, total sleep time, stage of rapid eye movement sleep, Epworth Sleepiness Score, triglycerides, total cholesterol, low-density lipoprotein, HDL, fasting blood glucose

*AHI* apnea/hypopnea index, *PRTS* percentage of recording time spent, *HDL* high-density lipoprotein

*P < 0.05, the results are statistically significant

## Discussion

This study was the first report on investigation of Sestrin2 in OSA, and the results showed that the Sestrin2 level elevated in OSA and decreased after nCPAP treatment. In addition, Sestrin2 was positively correlated with AHI, ODI, and oxygen saturation < 90% PRTS. Most importantly, the multiple regression analysis demonstrated that Sestrin2 was associated with AHI and oxygen saturation < 90% PRTS. It suggested that Sestrin2 played an important role in OSA.

Several authors [12, 13, 18–20] suggested that hypoxia, stress, or inflammation led to a growth of directed autophagy of Kelch-like ECH-associated protein 1 (Keap1), thereby decomposed the key regulator of oxidative genes, nuclear erythroid-related factor 2 (Nrf2). The production and accumulation of ROS inhibited by Nrf2 will lead to an increase in Sestrin2 [21, 22]. Moreover, hypoxia, stress, or inflammation will activate the mTORC1. Sestrin2, as a leucine sensor, is significantly increased in mTORC1 activation [23–25]. It was reported that hypoxia, oxidative stress, and inflammation recurred in OSA [26–28]. In present study, Sestrin2 was positively correlated with ODI and oxygen saturation < 90% PRTS. In addition, the multiple regression analysis showed that Sestrin2 was associated with oxygen saturation < 90% PRTS. Therefore, the increasing of urinary Sestrin2 might be triggered by intermittent hypoxia in OSA.

It was reported that Sestrin2 was a conserved antioxidant protein, and could be activated under pressure to protect cells from oxidative stress [29]. Essler et al. [30] suggested that Sestrin2 upregulation mediated by hypoxia was beneficial to counteracting the production of ROS, and ROS frequently accumulated and increased due to CIH in OSA [4]. Consequently, Sestrin2 might play an advantageous role in anti-oxidative stress in OSA.

It was reported that nCPAP could significantly reduce ODI and oxygen saturation < 90% PRTS in patients with OSA, and improve the hypoxia of OSA during sleep [31, 32]. In addition, the nCPAP could improve the oxidative stress and inflammatory response [32, 33]. In this study, the Sestrin2 reduced with the nCPAP treatment. Hypoxia, which might result in the elevation of Sestrin2, relieved after nCPAP treatment, thus the Sestrin2 decreased.

It was reported that central adiposity was associated with OSA [34], and it could predict the onset of OSA [35]. In present study, there was no relationship between waist-to-hip ratio and Sestrin2, which meant that the central adiposity had no effect on the change of Sestrin2.

Moreover, sleep architecture impairment of OSA might affect some factors, such as TNF-α [36]. However, in this study, Sestrin2 was not related to REM, NREM sleep, arousal index, and total sleep time, which meant that sleep architecture impairment of OSA did not contribute to increasing Sestrin2 level.

It was reported that there was a significant positive correlation between Sestrin2 and HDL [37]. The HDL played an antioxidant role [38], and the HDL levels were positively correlated with obesity status [39, 40]. Moreover, Sestrin2 regulated the PERK-c/EBPβ pathway by mediating mTORCI [41, 42], and inhibited the ROS-mediated p38 MAPK activation and interfered the expression of uncoupling protein 1 (Ucp1), subsequently resulting in the decrease of heat production and the increase of adipose tissue, which led to obesity [43, 44]. In addition, HDL was reduced in inflammatory conditions [45], while Sestrin2 could inhibit TLR-mediated inflammatory responses in macrophages [46]. Accordingly, it might be the obesity and inflammation that caused the association between Sestrin2 and HDL in this study.

However, there was a main limitation that the present study was not randomized; Small sample size was also a limitation. In addition, another limitation was that we used Auto-CPAP titration under PSG for pressure titration instead of the standard CPAP titration, though it was suggested that Auto-CPAP titration under PSG was also effective.

## Conclusion

Urinary Sestrin2 is involved in OSA, and is closely related to the severity of OSA. It decreases with nCPAP treatment. Accordingly, urinary Sestrin2 is a paramount marker of OSA severity.
